# Anxiety Symptom Burden at the Time of Forensic Evaluation in Child Victims of Sexual Assault: A Prospective Study Using the Beck Anxiety Inventory †

**DOI:** 10.3390/diagnostics16193192

**Published:** 2026-10-01

**Authors:** Kenan Kaya, Ahmet Aygün, Fatma Çam Aygün, Tuğçe Koca Yavuz

**Affiliations:** 1 Department of Forensic Medicine, Faculty of Medicine, Çukurova University, 01330 Adana, Türkiye; k_kaya_71@hotmail.com (K.K.); ahmet.aygun83@gmail.com (A.A.); 2 Adana Forensic Medicine Group Presidency, Council of Forensic Medicine, Ministry of Justice, 01060 Adana, Türkiye; drfatmacam@gmail.com; 3 Department of Forensic Medicine, Koç University Hospital, 34010 Istanbul, Türkiye

**Keywords:** Beck Anxiety Inventory, anxiety, child sexual abuse, child victim, forensic examination, trauma-informed care, child maltreatment

## Abstract

**Background/Objectives:** Research on child sexual abuse has largely addressed long-term sequelae, whereas anxiety at the time of forensic evaluation has rarely been quantified. This prospective study measured anxiety symptoms in child victims of sexual assault at forensic evaluation and identified the associated sociodemographic and incident-related factors. **Methods:** Children aged 6 to 17 years referred on suspicion of sexual assault and undergoing genital examination at a university forensic medicine department (Adana, Türkiye; May 2024 to May 2025) were evaluated prospectively. The Beck Anxiety Inventory (BAI) was administered immediately before examination. A proportional-odds ordinal regression model was fitted, with sensitivity analyses restricted to adolescents (12 to 17 years) and robustness analyses (bootstrap resampling, outcome dichotomisation and Firth penalization). **Results:** Of 104 children (82.7% female), 35 (33.7%) had severe anxiety. After adjustment, age group (aOR 1.95, 95% CI 1.05 to 3.65), penetration (aOR 5.16, 2.02 to 13.22) and disclosure to a formal recipient (aOR 2.48, 1.09 to 5.67) remained associated with anxiety. When restricted to adolescents, penetration (aOR 5.60) and formal disclosure (aOR 2.50) persisted while age lost significance (aOR 2.08); both were confirmed in bootstrap, dichotomised and Firth-penalised models. **Conclusions:** One third of children carried a severe anxiety burden at forensic evaluation, associated most strongly with penetration and formal disclosure and less consistently with older age, which may be partly artefactual. Without a comparison group, these exploratory findings describe associations rather than causal relationships and support routine psychiatric assessment and psychological support at forensic evaluation.

## 1. Introduction

Child abuse is defined by the World Health Organization as any form of behavior, neglect, or violation of rights, perpetrated intentionally or through negligence by an adult, society, or the state, that threatens a child’s physical and mental health, life, and developmental integrity. Within this framework, abuse is regarded as a set of non-accidental and preventable acts or omissions, committed by the person or persons responsible for the child’s care, that harm or have the potential to harm the child’s physical and psychological well-being and that adversely affect the child’s physical, emotional, cognitive, or sexual development [1].

From a forensic medicine perspective, child maltreatment is examined under four principal categories: physical abuse, sexual abuse, emotional abuse, and neglect [1,2,3]. This classification facilitates the systematic and holistic analysis of cases in both clinical evaluation processes and legal investigations [4].

Owing to its high global prevalence and severe consequences, child abuse is considered a major public health and forensic medicine concern [1,5]. According to recent reports by UNICEF, approximately two-thirds (around 66%) of children in the Europe and Central Asia region are subjected to violent discipline at home, in the form of physical punishment and/or psychological aggression. This finding highlights the cross-cultural prevalence of violence against children [6].

When country-specific data are examined, child abuse and neglect in the United Kingdom appear to represent a substantial mortality and morbidity burden. According to NSPCC data, an annual average of 56 children die from assault or undetermined intent based on a five-year average, with infants under one year of age constituting the highest-risk group [7]. Furthermore, approximately 1% of adults aged 18 to 74 years (481,000 individuals) are reported to have experienced physical neglect before the age of 16 nationwide [8]. Furthermore, early childhood has been reported to constitute a period of higher risk for abuse [9]. According to recent WHO data, six out of every ten children under the age of five regularly experience physical punishment and/or psychological violence at the hands of their parents and caregivers [10].

Data from the United States similarly underscore the magnitude of the problem. According to the most recent Child Maltreatment 2023 report published by the U.S. Department of Health and Human Services, more than three million reports of suspected child abuse and neglect are received annually by Child Protective Services (CPS) agencies [11]. The majority of these cases involve neglect, followed by physical abuse (17%) and sexual abuse (10.6%). Furthermore, approximately 2000 deaths related to child abuse and neglect are recorded each year, with 44% of these fatalities occurring in children under one year of age [12].

These findings clearly demonstrate that child abuse represents a major forensic and social problem with serious consequences in terms of both morbidity and mortality, and that addressing it effectively requires a multidisciplinary approach.

Epidemiological studies conducted in Türkiye indicate that the sexual abuse of children has reached a notable prevalence. Research in this area has shown that the rate of exposure to sexual abuse during childhood ranges between 9% and 18% [12,13,14]. A large-scale study conducted in Türkiye, which included 1785 child victims of sexual abuse, reported that 81.8% of cases were female and 18.2% were male, with a mean age of 12.7 ± 3.1 years (range 2 to 17) for girls and 10.7 ± 3.2 years (range 2 to 17) for boys [15].

Data from the Turkish Statistical Institute (TurkStat), an important source for epidemiological assessments, also support this picture. According to the 2024 “Statistics on Children Received or Brought to Security Units” published by the Turkish Statistical Institute (TurkStat), 279,620 children were recorded as having been referred to security units as victims during that year. Of these, 86.1% were classified as victims of crime, and 10.8% of the child crime victims were reported to have been victims of sexual offenses [16]. Taken together, these data indicate that childhood sexual abuse has a substantial prevalence in Türkiye, that girls are at particularly higher risk, and that a considerable proportion of cases are reflected in the judicial and healthcare systems.

The four-category classification of maltreatment is also reflected in the DSM-5 and, similarly to its predecessor DSM-IV, child abuse is addressed within the scope of “V codes.” These codes do not constitute a direct diagnosis of a mental disorder; rather, they identify conditions that warrant clinical attention and intervention. According to the DSM-5, child sexual abuse is defined within a broad framework that encompasses any behavior performed by a perpetrator for the purpose of sexual gratification. In this context, the perpetrator may be a parent, caregiver, or any individual with responsibility over the child, as well as other persons in contact with the child. Such behaviors include contact-based acts such as genital contact, penetration, rape, incest, sodomy, and exhibitionism, as well as non-contact behaviors carried out through threat, coercion, deception, manipulation, or pressure. Similarly, the ICD-11 defines child sexual abuse as any sexual act in which a child is involved for the purpose of an adult’s sexual gratification. This includes oral, vaginal, or anal penetration; contact made directly or through clothing; and circumstances in which the child is coerced, seduced, threatened, or manipulated into engaging in sexual behavior. Furthermore, situations in which a child is forced to appear in sexually explicit images, exposed to pornographic material, or directed toward sexual contact with third parties are also considered within this scope. These definitions demonstrate that child sexual abuse is not limited to physical contact; rather, it encompasses a broad spectrum of behaviors that threaten the sexual integrity and psychosocial development of the child [2].

Childhood is a critical period during which physical and psychological development accelerates and self-perception, along with patterns of social behavior, takes shape. For this reason, traumatic experiences encountered during this period may exert lasting and profound effects not only on physical health but also on the psychological structure. The pre-trauma mental status and psychiatric background of the victim are among the variables that have been associated with the severity of the trauma’s effects. While some children with more developed coping skills appear to experience the consequences of distressing events to a more limited extent, individuals with more fragile personality structures are known to exhibit more pronounced psychopathological findings following abuse [17]. Indeed, in a study conducted on adolescents aged 14 to 18 years who had been subjected to sexual abuse, those who exhibited psychiatric symptoms were classified as “vulnerable” and those who did not as “resilient”; individuals in the vulnerable group were found to have more prominent prior psychiatric histories, as well as significantly higher levels of depression and anxiety [18].

Although cases of sexual abuse are most commonly referred to child and adolescent psychiatry clinics by families on suspicion of abuse or as part of judicial proceedings, findings suggestive of abuse may also be identified during clinical evaluations of children presenting for unrelated reasons [19]. A substantial proportion of referrals are prompted by the psychological and somatic complaints that result from abuse [20]. Although there is no single psychiatric diagnosis specific to sexual abuse, it has been reported that these cases are at high risk of developing mental disorders and that their likelihood of receiving various psychiatric diagnoses increases over the long term [21]. Indeed, a 43-year cohort study found that the rate of mental health service utilization among individuals exposed to sexual abuse during childhood was 3.6 times higher than that of healthy controls. The same study demonstrated that mood disorders, anxiety disorders, post-traumatic stress disorder (PTSD), alcohol and substance use disorders, eating disorders, somatization disorders, and borderline, antisocial, and dissociative personality disorders occurred significantly more frequently in these individuals [22].

Data on victims of sexual abuse evaluated at forensic units in Türkiye in 2015 also support these findings. Accordingly, the diagnostic criteria for major depression were met in 45.9% of cases, PTSD in 31.7%, acute stress disorder in 11.5%, anxiety disorder in 1.1%, and conversion disorder in 1.1%. Moreover, although no psychiatric diagnosis was established in 24.2% of cases, intense emotional reactions such as anger and guilt, as well as sleep disturbances, were frequently observed [23]. The literature indicates strong associations between childhood sexual abuse and major depression, bipolar disorder, generalized anxiety disorder, panic disorder, specific phobias, and PTSD [24,25,26]. Furthermore, early-life traumas have been emphasized as being associated with an increased risk of schizophrenia, reactive attachment disorder, eating disorders, personality disorders, and substance use disorders, and as constituting a significant predisposition to suicidal behavior [27].

The Beck Anxiety Inventory (BAI) is a self-report measurement instrument developed to quantitatively assess the severity of anxiety symptoms in individuals. The inventory was developed by Aaron T. Beck and colleagues in 1988 and was designed to differentiate the symptom pattern specific to anxiety from depressive symptoms. In this respect, the BAI is widely used as an assessment tool in both clinical and research settings [28]. The inventory consists of 21 items and evaluates the individual’s anxiety symptoms over the past week. Items are rated on a four-point Likert-type scale ranging from 0 to 3, with total scores ranging from 0 to 63. Higher scores indicate greater severity of anxiety [29].

A review of the literature reveals that studies examining the psychological status of child victims of sexual abuse have largely focused on the long-term effects of trauma. In contrast, the psychological responses that emerge during the early stages of the judicial process, and acute anxiety levels in particular, have been addressed in only a limited number of studies. The forensic evaluation process is a stressful experience that may trigger the re-experiencing of traumatic events for the child and that carries the risk of secondary victimization. The scarcity of studies investigating the relationship between anxiety experienced during this process and the characteristics of the incident, as well as sociodemographic variables, is noteworthy. However, the period surrounding the forensic examination is a critical phase during which the child both re-experiences the trauma and confronts uncertainty and fear. This points to a knowledge gap regarding the psychological effects of the forensic examination process on children and the development of trauma-informed approaches [30].

To our knowledge, no previous study has quantified anxiety symptoms in child victims of sexual assault at the point of forensic genital examination using a standardised instrument, reported the full distribution of anxiety severity in this setting, and examined which characteristics remain associated with anxiety after mutual adjustment. The aim of this study was therefore to assess anxiety levels in child victims of sexual assault in Türkiye at the time of forensic examination using the Beck Anxiety Inventory, and to examine the relationships between these levels and individual variables such as age and sex, as well as incident-specific factors such as the relationship to the perpetrator, the presence of penetration, physical findings, and time elapsed before presentation. Furthermore, the findings are intended to contribute to the literature and to inform the development of judicial processes in line with child-friendly and trauma-informed approaches.

## 2. Materials and Methods

### 2.1. Study Design and Participants

This study was designed as a prospective, descriptive investigation. Children referred by judicial authorities to the Department of Forensic Medicine, Balcalı Hospital, Çukurova University Faculty of Medicine, on suspicion of sexual assault between 11 May 2024 and May 2025, and who underwent forensic genital examination, were included in the study.

Cases were evaluated with respect to age, sex, the perpetrator’s relationship to the victim, educational status, cohabitants, the presence and type of penetration, anogenital findings, accompanying physical injury, alcohol or substance influence at the time of the incident, history of a previous anogenital examination, time elapsed between the incident and the examination, history of psychiatric treatment, and the recipient of the child’s first disclosure. Anxiety was measured using the Beck Anxiety Inventory.

Age was analysed in three groups: 6 to 11 years, 12 to 14 years and 15 to 17 years. These boundaries are mutually exclusive.

Penetration was defined as any reported anal, oral or vaginal penetration and was recorded as a binary variable (present or absent) for the primary analysis; penetration type was recorded separately, and cases involving more than one type were coded for each type present.

Alcohol or substance influence at the time of the incident was recorded as present when the child reported having consumed alcohol or a psychoactive substance in the period immediately preceding the alleged assault, or when this was documented in the accompanying judicial file. The variable denotes acute exposure in relation to the index incident and does not indicate habitual use, dependence or a diagnosed substance-use disorder. Information was obtained from the child’s account during the forensic interview and from the case file, without toxicological confirmation.

History of psychiatric treatment denotes any psychiatric treatment received before the index incident, ascertained from the child’s account and the accompanying file. Neither the nature of the diagnosis nor whether treatment was ongoing at the time of assessment was recorded.

### 2.2. Inclusion and Exclusion Criteria

Children aged 6 to 17 years who were referred by judicial authorities on suspicion of sexual assault and underwent forensic genital examination during the study period were eligible for inclusion. A lower age limit of 6 years was set in the study protocol, since the Beck Anxiety Inventory requires a level of symptom recognition and one-week recall that is not developmentally available to younger children. Written informed consent was obtained from the legal guardian and verbal assent from the child, in accordance with the child’s developmental level.

Exclusion criteria were:

1. Age under 6 years or 18 years and over.
2. Refusal by the child to participate, or absence of consent from the legal guardian.
3. Cognitive or communicative capacity insufficient to understand and answer the items of the Beck Anxiety Inventory.
4. Administration of sedation, general anaesthesia or any agent affecting the level of consciousness before the examination.
5. Haemodynamic instability, life-threatening trauma, or any condition requiring emergency medical intervention.
6. Clinical urgency requiring immediate genital examination, leaving insufficient time for administration of the inventory.
7. A communication barrier preventing standard administration of the inventory, such as a language barrier without available interpreter support.
8. Incomplete or invalid completion of the Beck Anxiety Inventory.
9. Any other psychological assessment or intervention carried out before the genital examination that could have influenced the outcome measure.

### 2.3. Beck Anxiety Inventory and Its Administration

The Beck Anxiety Inventory (BAI) is a 21-item self-report instrument developed by Beck et al. to quantify the severity of anxiety symptoms experienced over the preceding week [28]. Items are rated on a four-point Likert-type scale from 0 to 3, giving total scores between 0 and 63, with higher scores indicating more severe anxiety [29]. Total scores were classified as minimal (0 to 7), mild (8 to 15), moderate (16 to 25) or severe (26 to 63) anxiety. These labels denote BAI symptom-severity categories based on total scores and do not represent a clinical diagnosis of an anxiety disorder.

The inventory was administered to every participant immediately before the forensic genital examination, in a private room, by a forensic medicine specialist. No accompanying adult was present during administration, in either age group. For children aged 6 to 11 years, the investigator read each item aloud and, where a child did not understand a symptom description, provided a clarification in age-appropriate language, taking care that the clarification did not indicate or favour any response option; the child then selected the response. Children aged 12 years and over completed the inventory independently after receiving standard instructions.

The BAI quantifies anxiety symptoms experienced over the preceding seven days. Administering the inventory immediately before the forensic examination therefore captures the child’s anxiety state during the week encompassing the alleged incident, the referral and the judicial process, and does not isolate anxiety attributable specifically to the impending examination. Throughout this manuscript, BAI scores are interpreted as a measure of anxiety symptom burden at the time of forensic evaluation rather than as examination-induced anxiety.

### 2.4. Statistical Analysis

Statistical analyses were performed using IBM SPSS Statistics version 22.0. Categorical variables are expressed as numbers and percentages.

In all contingency tables, percentages are reported as row percentages, that is, the proportion of children within each sociodemographic or clinical group who fell into each anxiety category. This denominator was chosen because the quantity of clinical interest is the prevalence of each anxiety category within an exposure group, rather than the composition of each anxiety category.

Associations between anxiety category and each variable were assessed using Pearson’s chi-square test. Fisher’s exact test was used for 2 by 2 tables, and the Fisher–Freeman–Halton exact test (Monte Carlo estimation, 20,000 replicates) was used for larger tables in which more than 20% of expected cell counts were below five.

To assess whether the associations observed in bivariate analysis were independent of one another, a multivariable ordinal logistic regression model was fitted with the four-level BAI category as the ordered outcome. Predictors were age group (entered as an ordinal term), educational status, presence of penetration, alcohol or substance influence at the time of the incident, history of psychiatric treatment, and whether the child’s first disclosure was to a formal recipient (teacher, law-enforcement officer or healthcare professional) rather than to family, relatives or friends. The proportional-odds assumption was examined by fitting separate binary logistic models at each of the three cut-points and testing the equality of coefficients across cut-points using a Wald-type statistic. The assumption was supported both for individual predictors (all *p* values 0.089 or greater) and overall (chi-square 13.65, df 12, *p* = 0.323); a proportional-odds model was therefore retained. Odds ratios are reported with 95% confidence intervals, and model fit is summarised by the likelihood-ratio statistic and McFadden’s pseudo R-squared.

The variables examined were defined prospectively in the study protocol; however, no formal hypotheses regarding the direction or magnitude of individual associations were prespecified, and the bivariate analyses should therefore be regarded as exploratory. Sixteen bivariate comparisons were performed. No formal correction for multiple testing was applied, as such correction would be inappropriate to the exploratory nature of these analyses; instead we note that at a nominal significance level of 0.05, approximately one significant result would be expected by chance among sixteen tests. Interpretation therefore emphasises the multivariable model and the magnitude and precision of effect estimates rather than statistical significance alone.

To address the potential influence of the age-dependent difference in BAI administration, a post hoc sensitivity analysis was performed by refitting the multivariable model in the subgroup of participants aged 12 to 17 years, among whom the inventory was self-completed under uniform conditions, and by examining whether the association between penetration and anxiety persisted within this subgroup. The robustness of the multivariable estimates was further examined by refitting the model as a binary logistic regression after collapsing the outcome into moderate-to-severe (BAI ≥ 16) versus minimal-to-mild anxiety, by deriving percentile 95% confidence intervals from 2000 bootstrap resamples, and by repeating the analysis under an alternative dichotomisation (severe versus non-severe) and with a Firth penalised logistic model to reduce small-sample bias. The percentile bootstrap confidence intervals (boot package) and the Firth penalised model (logistf package) were computed in R (version 4.3.3; R Foundation for Statistical Computing, Vienna, Austria); the remaining sensitivity analyses were performed in IBM SPSS Statistics version 22.0.

The level of statistical significance was set at *p* < 0.05.

### 2.5. Ethical Approval

This study was approved by the Research Ethics Committee of Çukurova University Faculty of Medicine on 10 May 2024 (Decision No. 144/6) and was conducted in accordance with the Declaration of Helsinki.

Generative artificial intelligence (GenAI) disclosure: During the preparation of this manuscript, a generative artificial intelligence (GenAI) tool, namely Claude (Anthropic, San Francisco, CA, USA; Claude Opus 5), was used to improve the clarity, grammar and linguistic quality of the English text, and to format the manuscript in accordance with the journal template. All analyses were verified by the authors against the source dataset. The tool was not involved in the study design, data acquisition, interpretation of the findings or any scientific decision-making process. All scientific concepts, analyses, conclusions and manuscript revisions were independently evaluated and approved by the authors, who assume full responsibility for the accuracy, validity and integrity of the work.

## 3. Results

A total of 104 children were included in the study. Of these, 82.7% (*n* = 86) were female and 17.3% (*n* = 18) were male. Examination of the age distribution revealed that 13.5% (*n* = 14) were aged 6 to 11 years, 37.5% (*n* = 39) 12 to 14 years, and 49.0% (*n* = 51) 15 to 17 years. According to the Beck Anxiety Inventory, minimal anxiety was identified in 20.2% of children (*n* = 21), mild anxiety in 25.0% (*n* = 26), moderate anxiety in 21.2% (*n* = 22), and severe anxiety in 33.7% (*n* = 35).

All percentages reported below and in the tables are row percentages, that is, the proportion of children within each group who fell into a given anxiety category.

### 3.1. Sociodemographic Characteristics

Anxiety category differed significantly across age groups (*p* < 0.001). Severe anxiety was recorded in 27 of 51 children aged 15 to 17 years (52.9%) and in 8 of 39 children aged 12 to 14 years (20.5%), while no child aged 6 to 11 years was classified in the severe category (Table 1).

No significant association was identified between sex and anxiety category (*p* = 0.122) (Table 2).

When educational status was evaluated, severe anxiety was recorded in 19 of 36 children who had discontinued their education (52.8%) and in 16 of 68 children who were attending school (23.5%); this difference was statistically significant (*p* = 0.013) (Table 3).

Children were classified according to cohabitants as living with both parents, with the mother only, with the father only, with relatives, or in a residential care institution. No significant association was identified between cohabitation status and anxiety category (*p* = 0.360) (Table 4).

### 3.2. Incident-Related Characteristics

Penetration was reported in 77 children (74.0%) and was absent in 27 (26.0%). Severe anxiety was recorded in 34 of the 77 children with penetration (44.2%) and in 1 of the 27 without penetration (3.7%). This association was highly significant (*p* < 0.001) (Table 5).

Among the 77 children in whom penetration occurred, the type of penetration was vaginal only in 40, anal and vaginal in 16, anal only in 11, oral and vaginal in 6, anal and oral in 3, and all three types in 1. Vaginal penetration was therefore involved in 63 of the 77 cases with penetration (81.8%), corresponding to 60.6% of the whole sample. When the analysis was restricted to children in whom penetration had occurred, no penetration subtype was significantly associated with anxiety category (Table 6).

Values are n (row %). Categories are not mutually exclusive: 26 children experienced more than one type of penetration. Vaginal and oral comparisons were tested with the Fisher–Freeman–Halton exact test; the anal comparison with Pearson’s chi-square test.

Alcohol or substance influence at the time of the incident was recorded in 30 children (28.8%). Severe anxiety was recorded in 16 of these 30 children (53.3%) and in 19 of the 74 children without such exposure (25.7%); the association was significant in unadjusted analysis (*p* = 0.019) (Table 7).

A history of psychiatric treatment before the index incident was identified in 33 children (31.7%). Severe anxiety was recorded in 17 of these 33 children (51.5%) and in 18 of the 71 children without such a history (25.4%); the association was significant in unadjusted analysis (*p* = 0.004) (Table 8).

### 3.3. Disclosure

The recipient of the child’s first disclosure was the nuclear family in 41 cases (39.4%), a teacher in 20 (19.2%), a friend in 19 (18.3%), a law-enforcement officer in 13 (12.5%), a relative in 9 (8.7%) and a healthcare professional in 2 (1.9%). Anxiety category was significantly associated with the recipient of first disclosure (*p* = 0.019). Severe anxiety was recorded in 8 of 13 children who disclosed to a law-enforcement officer (61.5%), in 9 of 20 who disclosed to a teacher (45.0%), in 6 of 19 who disclosed to a friend (31.6%), in 10 of 41 who disclosed to a member of the nuclear family (24.4%) and in 1 of 9 who disclosed to a relative (11.1%) (Table 9).

### 3.4. Variables Not Associated with Anxiety Category

Five further variables recorded in the study protocol showed no statistically significant association with anxiety category. They are reported in full for completeness (Table 10).

### 3.5. Multivariable Analysis

The proportional-odds assumption was supported for the ordinal model, both for individual predictors (all *p* values 0.089 or greater) and overall (chi-square 13.65, df 12, *p* = 0.323).

In the adjusted model, three variables remained independently associated with anxiety category: age group (aOR 1.95 per one-category increase, 95% CI 1.05 to 3.65, *p* = 0.035), the presence of penetration (aOR 5.16, 95% CI 2.02 to 13.22, *p* = 0.001) and first disclosure to a formal recipient (aOR 2.48, 95% CI 1.09 to 5.67, *p* = 0.031). The associations with educational status, alcohol or substance influence and prior psychiatric treatment, all significant in unadjusted analysis, did not persist after adjustment (Table 11). Given the sample size and the number of predictors, these adjusted associations should be regarded as exploratory and hypothesis-generating rather than as definitive independent predictors.

Three further variables assessed in unadjusted analysis were not carried into the multivariable model: male sex (cOR 0.35, 95% CI 0.14 to 0.92, *p* = 0.033), a previous anogenital examination (cOR 2.33, 95% CI 0.99 to 5.49, *p* = 0.052) and not living with both parents (cOR 2.10, 95% CI 1.04 to 4.24, *p* = 0.039).

### 3.6. Sensitivity and Robustness Analyses

To assess whether the age-related association reflected the difference in BAI administration between age groups, the multivariable model was refitted in the subgroup of participants aged 12 to 17 years (n = 90), among whom the inventory was self-completed under uniform conditions. In this subgroup, the presence of penetration (aOR 5.60, 95% CI 1.98 to 15.82, *p* = 0.001) and first disclosure to a formal recipient (aOR 2.50, 95% CI 1.07 to 5.80, *p* = 0.033) remained independently associated with anxiety category, whereas the association with age was attenuated and no longer statistically significant (aOR 2.08, 95% CI 0.89 to 4.85, *p* = 0.090). Penetration was also associated with anxiety within this subgroup before adjustment (crude OR 7.50, 95% CI 2.79 to 20.19, *p* < 0.001). The attenuation of the age association once the youngest, investigator-assisted group was excluded is consistent with this association being at least partly attributable to the difference in administration mode.

The stability of the multivariable estimates was examined further (Table S1). Percentile bootstrap 95% confidence intervals (2000 resamples) excluded unity for all three predictors that were significant in the main model (age 1.05 to 4.18; penetration 2.14 to 15.77; formal disclosure 1.11 to 6.15). When the outcome was dichotomised into moderate-to-severe (BAI ≥ 16) versus minimal-to-mild anxiety, the associations with penetration (aOR 14.86, 95% CI 3.37 to 65.55, *p* < 0.001) and formal disclosure (aOR 4.35, 95% CI 1.35 to 14.05, *p* = 0.014) persisted, and a Firth penalised model, which reduces small-sample bias, yielded concordant but less extreme estimates (penetration aOR 11.13, 95% CI 3.22 to 51.72; formal disclosure aOR 3.76, 95% CI 1.31 to 12.21). Under an alternative dichotomisation (severe [BAI ≥ 26] versus non-severe), penetration remained associated with anxiety (aOR 10.80, 95% CI 1.25 to 93.23, *p* = 0.031), whereas the association with formal disclosure was attenuated (*p* = 0.254), indicating that disclosure route was related to the broader gradient of anxiety severity rather than specifically to the most severe category. Across all specifications, the association with penetration was the most consistent, that with formal disclosure was largely stable, and that with age was the least stable.

## 4. Discussion

Child sexual abuse is associated with a broad range of psychological difficulties, and anxiety symptoms may occur both in the immediate aftermath of an assault and over the longer term [31,32,33,34]. However, the severity and expression of psychological symptoms vary considerably between individuals, and available evidence suggests that the characteristics of the assault, individual vulnerabilities, developmental stage, and social context may all contribute to this heterogeneity [31,32,33,34,35,36,37]. Against this background, the present study examined anxiety symptoms measured at the time of forensic evaluation in 104 children referred following an alleged sexual assault. The main finding was that anxiety symptoms were common in this forensic setting, with approximately one third of participants classified in the severe category and a further one fifth in the moderate category. Throughout, “severe” refers to a BAI severity category rather than a diagnosed anxiety disorder; this distinction is particularly important in a forensic setting, where symptom scores could be over-interpreted in clinical or legal contexts. Moreover, because the study did not include a comparison group, the clinical significance of this prevalence cannot be established: it cannot be determined whether these anxiety levels exceed those of children of similar age in the general population, of sexually abused children who do not undergo forensic examination, or of children undergoing other medical evaluations. The one-third prevalence of severe-range scores should therefore be regarded as descriptive rather than as evidence of examination-specific or abuse-specific anxiety. In adjusted analyses, older age, the presence of penetration, and first disclosure through a formal channel remained associated with a higher anxiety category. These findings should, however, be interpreted as exploratory associations rather than evidence of independent causal effects, given the sample size, measurement limitations, cross-sectional assessment of anxiety, and potential residual confounding. Because sixteen bivariate comparisons were performed and only three variables remained associated after adjustment, the discussion below focuses primarily on these adjusted findings (age, penetration, and formal disclosure); variables that were significant only in unadjusted analysis (educational status, substance influence, and prior psychiatric treatment) are discussed more briefly and interpreted with correspondingly greater caution.

### 4.1. Age and Anxiety

Older age was associated with a higher anxiety category in both unadjusted and adjusted analyses. Developmental stage may plausibly influence the way children perceive, interpret, and report threatening experiences. Adolescents may have greater cognitive awareness of the interpersonal and social implications of sexual assault and may anticipate consequences related to family relationships, stigma, disclosure, legal proceedings, or future sexual and interpersonal functioning. The previous literature has similarly identified developmental and individual differences in psychological responses following childhood sexual abuse [31,32,33,34,37].

Nevertheless, the observed age association requires particular caution. Children aged 6–11 years in the present study received investigator-assisted administration of the Beck Anxiety Inventory (BAI), whereas older participants completed the instrument independently. These procedures are not psychometrically equivalent, even though clarification was non-directive. Consequently, the association between age and anxiety may reflect a combination of genuine developmental differences and differences in assessment mode. This issue is especially important because age was one of the variables that remained associated with anxiety after adjustment. The present data cannot determine the extent to which administration mode contributed to this finding. Consistent with this, a sensitivity analysis restricted to participants aged 12 to 17 years, among whom administration was uniform, showed that the age association was attenuated and no longer significant, whereas the associations with penetration and formal disclosure persisted (Section 3.6). This pattern supports the interpretation that the age effect observed in the full sample is at least partly artefactual. Future studies should use age-appropriate instruments with validated administration procedures that are consistent across developmental groups.

### 4.2. Penetration and Anxiety

The presence of penetration was associated with higher anxiety category in both unadjusted and adjusted analyses, whereas the specific type of penetration was not associated with anxiety after accounting for the presence of penetration. This distinction is important because it suggests that, within the limitations of the present dataset, the occurrence of penetration rather than a particular anatomical type of penetration was the more relevant marker of anxiety severity.

Several mechanisms may plausibly contribute to this association. Penetrative assault may involve a greater perceived violation of bodily integrity and may be accompanied by an acute subjective appraisal of threat, fear, helplessness, shame, or self-blame. Previous studies have described the relevance of abuse-related characteristics, attributional processes, shame, and social responses to subsequent psychological adjustment [38,39,40,41,42,43]. However, these mechanisms were not directly measured in the present study. They should therefore be regarded as possible explanations rather than demonstrated mediating pathways.

The association should also not be interpreted as evidence that penetration causes higher anxiety symptoms. Anxiety was assessed at a single time point, and the study did not include measures of perceived threat, fear, shame, attributional style, or other potential mediators. Furthermore, penetration was strongly patterned by age in the present sample. Although the association persisted after adjustment, residual confounding by unmeasured characteristics of the assault, the child’s circumstances, or differences in assessment across age groups cannot be excluded. In particular, the presence of penetration may function partly as a marker of overall assault severity, standing in for unmeasured characteristics such as the degree of coercion, physical force, chronicity, or perceived threat, rather than acting as a direct and independent determinant of anxiety. The observed association should therefore be read as consistent with, but not specific to, a direct effect of penetration itself.

### 4.3. Alcohol or Substance Influence at the Time of the Incident

Alcohol or psychoactive substance influence at the time of the alleged incident was associated with higher anxiety category in the unadjusted analysis but not after adjustment (aOR 1.88, 95% CI 0.72 to 4.92, *p* = 0.200). Because this variable reflects an acute circumstance of the incident rather than habitual substance use or a longer-term consequence of abuse, it is not directly comparable with much of the previous literature, which has examined substance use as a later correlate of childhood sexual abuse [44,45,46,47,48]. Alcohol-involved sexual assault occurs more commonly in later adolescence [49], so this variable may function primarily as a marker of an older, differently exposed subgroup rather than as an independent determinant of anxiety. The present data cannot distinguish between these possibilities, and this association should not be over-interpreted.

### 4.4. Previous Psychiatric Treatment

A history of psychiatric treatment before the alleged incident was likewise associated with higher anxiety category in the unadjusted analysis but not after adjustment (aOR 1.28, 95% CI 0.51 to 3.22, *p* = 0.600), and thus provides no independent statistical support for such a relationship in this sample. Pre-existing psychiatric difficulty is a plausible vulnerability factor [50,51,52], but the variable is heterogeneous, and current psychotropic medication use, which could alter the expression of anxiety symptoms, was not recorded and represents a source of unmeasured confounding. The wide confidence interval indicates substantial uncertainty, and larger studies with structured psychiatric assessment and medication status would be needed to clarify any independent contribution.

### 4.5. Disclosure Route and Anxiety

The person to whom the child first disclosed the alleged incident showed an association with anxiety category. Children whose first disclosure was made to a formal recipient—defined in this study as a teacher, law-enforcement officer, or healthcare professional—were more likely to be in a higher anxiety category, and this association remained after adjustment (aOR 2.48, 95% CI 1.09–5.67).

This finding should not be interpreted as demonstrating that formal disclosure increases anxiety. The temporal relationship between disclosure and anxiety cannot be established because both were assessed within the same overall period. Several explanations are therefore possible. Children experiencing greater distress may be more likely to disclose outside the family, particularly when they require assistance or when the incident is identified through institutional or professional channels. Conversely, circumstances associated with formal disclosure—such as limited family support, greater visibility or severity of the assault, involvement of a family member, or involvement of judicial authorities—may themselves be associated with higher anxiety. It is also possible that subsequent formal procedures contribute to anxiety. The present design cannot distinguish among these explanations. In particular, this association should not be read as indicating that disclosure to a teacher, law-enforcement officer, or healthcare professional itself increases a child’s anxiety.

The distribution of disclosure recipients in our sample was broadly consistent with the previous literature indicating that adolescents may disclose unwanted sexual experiences to family members or peers, while disclosure to professionals may be less frequent [53]. In the present study, nuclear family members were the most frequent first recipients, followed by teachers and friends. The relatively frequent role of teachers may reflect their position as adults who interact regularly with adolescents outside the home and may identify concerns through school-based guidance and safeguarding processes. Findings from Turkish forensic samples have likewise highlighted the role of parents and other adults in disclosure and identification processes [54].

The clinical significance of the disclosure finding is therefore better understood as an indicator of case complexity and of the social and institutional context surrounding the child’s presentation, rather than as evidence for a causal effect of disclosure route on anxiety. Future longitudinal studies should assess anxiety before disclosure, after disclosure, and at subsequent stages of the forensic and judicial process to clarify the temporal relationship.

### 4.6. Other Clinical and Contextual Variables

Several other variables recorded in the study protocol, including the perpetrator’s relationship to the child, accompanying physical injury, anogenital findings, previous anogenital examination, and time elapsed between the alleged incident and forensic examination, were not significantly associated with anxiety category. These null findings should be interpreted cautiously rather than as evidence that these characteristics have no psychological relevance. The sample was relatively small for subgroup analyses, and the study was not powered specifically to detect modest associations.

The absence of an association between time to examination and anxiety is of particular interest. One might hypothesise that children examined shortly after the incident would report greater anxiety because of the temporal proximity of the event. However, the present findings do not support such an association. The relatively coarse categorisation of elapsed time and the absence of repeated measurements limit the conclusions that can be drawn. Anxiety may also change dynamically during the interval between the incident, disclosure, referral, judicial procedures, and examination, which cannot be captured by a single assessment.

### 4.7. Interpretation of the Multivariable Findings

The multivariable analysis should be regarded as exploratory. The study included 104 participants, and the smallest anxiety category contained only 21 children, while six predictors were entered into the model. This corresponds to approximately 3.5 observations in the smallest outcome category per predictor, substantially below commonly cited rules of thumb for stable regression modelling. Consequently, the adjusted estimates may be sensitive to the sample composition, and the confidence intervals indicate considerable uncertainty.

For this reason, the persistence of age, penetration, and formal disclosure in the adjusted model should not be interpreted as demonstrating robust independent determinants of anxiety severity. Rather, these variables identify associations that remained detectable after adjustment for the variables included in the model. Importantly, adjustment can only account for measured covariates; it cannot correct for measurement non-equivalence across age groups, unmeasured confounding, or the absence of information on current medication and other potentially relevant factors.

Taken together, the findings provide a descriptive signal that anxiety symptoms are substantial among children presenting for forensic genital examination and that anxiety category varies according to several characteristics of the child and the reported incident. The results are better viewed as hypothesis-generating than as estimates of causal effects. These interpretations are supported by the robustness analyses reported in Section 3.6: bootstrap resampling, outcome dichotomisation, and a Firth penalised model consistently confirmed the association with penetration and, to a lesser extent, with formal disclosure, whereas the age association was the least stable across specifications. Larger studies using validated paediatric anxiety measures, consistent administration procedures, longitudinal assessments, and prespecified statistical models are needed to determine whether the observed associations are reproducible.

### 4.8. Limitations

Several limitations should be considered when interpreting the findings.

First, the validity and comparability of the anxiety measure across age groups are important concerns. The BAI was originally developed and validated primarily in adult populations [28]. Although the psychometric properties of the Turkish version of the BAI have been demonstrated previously [55], the available Turkish validation study did not include children as young as six years, and the instrument has not been psychometrically validated for use in this youngest age group. Its use in the 6-to-11-year group therefore represents a measurement limitation. For these children the investigator read the items aloud and provided age-appropriate clarification to improve comprehensibility; this procedure was intended to aid comprehension and does not imply that the scale has been validated for use in children. In the present study, children aged 6–11 years received investigator-assisted administration, whereas older participants completed the instrument independently. Although clarification was non-directive, these procedures are not psychometrically equivalent. Because age was also associated with anxiety category, the observed age effect may therefore be partly influenced by differences in administration mode. This limitation cannot be resolved retrospectively. Future studies should use instruments specifically validated for children and adolescents and employ comparable administration procedures across developmental groups.

Second, the BAI measures symptoms experienced during the preceding week. Because the instrument was administered immediately before forensic examination, the resulting score cannot be interpreted as examination-specific anxiety. The measured symptoms may reflect the alleged assault, the period following disclosure, referral and judicial procedures, anticipation of the forensic examination, pre-existing anxiety, or a combination of these factors. No comparison group was included, and anxiety was not measured repeatedly before and after the examination. Therefore, the present study cannot determine the proportion of the measured anxiety attributable to any one of these components. A comparison group and repeated measurements would be required to isolate examination-related anxiety.

Third, although participants were prospectively recruited, the analyses concerning anxiety and its correlates are essentially cross-sectional because anxiety and the principal explanatory variables were assessed within the same overall assessment period. Consequently, temporal ordering and causal direction cannot be established. This is particularly relevant to the association between anxiety and disclosure route. The data cannot determine whether higher anxiety influenced the choice or circumstances of disclosure, whether formal disclosure occurred under circumstances associated with greater anxiety, or whether subsequent formal procedures contributed to anxiety.

Fourth, the sample size limits the stability and precision of the multivariable analysis. The study included 104 children, with only 21 participants in the smallest anxiety category, while six predictors were included in the ordinal logistic regression model. The resulting approximately 3.5 observations in the smallest outcome category per predictor indicate limited statistical information for the fitted model. The adjusted estimates should therefore be considered exploratory, and the relatively wide confidence intervals indicate substantial uncertainty. Additional statistical modelling cannot compensate for the limited information contained in the sample.

Fifth, potentially important confounding variables were not measured. In particular, current psychotropic medication use was not recorded, despite approximately one third of the children having a history of psychiatric treatment. Medication use could influence the severity of anxiety symptoms reported at assessment, but its prevalence and distribution across the study groups are unknown. Alcohol or psychoactive substance influence at the time of the alleged incident was determined from self-report and case-file information without toxicological confirmation. In addition, no structured psychiatric interview was performed to establish current or previous psychiatric diagnoses. These limitations restrict the ability to account comprehensively for pre-existing psychological characteristics and treatment effects.

Sixth, several potentially relevant psychological mechanisms were not measured. For example, perceived threat, fear, helplessness, shame, self-blame, social support, and coping responses may influence psychological adjustment following sexual assault [31,40,41,42,43,44,45]. Because these variables were not collected, the present study cannot determine whether they mediate or modify the associations observed for penetration, age, or disclosure route.

Seventh, several subgroups were small. In particular, only six children were living with the father alone, thirteen were residing in a residential care institution, and two first disclosed to a healthcare professional. Estimates for such small subgroups are inherently unstable and should not be interpreted individually. Similarly, the absence of statistically significant associations for several clinical variables may reflect limited power rather than evidence of no relationship.

Finally, the study was conducted in a single forensic medicine department and included children referred by judicial authorities who underwent forensic genital examination. The findings may therefore not generalise to all children who experience sexual abuse, including those who do not enter the judicial system, those evaluated in other clinical settings, or those who do not undergo genital examination. The sample also represents children presenting within a particular forensic and institutional context, which may influence both anxiety symptoms and disclosure patterns. Referral pathways, disclosure practices, forensic and judicial procedures, and cultural attitudes toward sexual abuse differ substantially across countries, and these differences may shape both which children present for forensic evaluation and the anxiety they experience. The present findings should therefore be interpreted within the specific Turkish medico-legal context and confirmed in other national and cultural settings before broader generalisation. Multicentre studies with larger samples, repeated assessments, age-appropriate validated instruments, consistent administration procedures, structured psychiatric assessment, medication data, and appropriate comparison groups are needed to establish the reproducibility and clinical significance of these findings.

## 5. Conclusions

In this prospective study of 104 children referred following an alleged sexual assault, anxiety symptoms measured at the time of forensic evaluation were substantial, with approximately one third of participants classified in the severe category and a further one fifth in the moderate category.

In unadjusted analyses, higher anxiety categories were associated with older age, discontinued education, penetration, alcohol or psychoactive substance influence at the time of the incident, previous psychiatric treatment, and disclosure through a formal channel. After adjustment, associations with age, the presence of penetration, and formal disclosure remained, whereas the associations with educational status, substance influence, and previous psychiatric treatment did not. The age association, however, may partly reflect the difference in BAI administration mode between age groups. The type of penetration was not associated with anxiety category after accounting for the presence of penetration.

These findings should be interpreted as exploratory associations rather than causal effects. In particular, the limited sample size and model stability, the different BAI administration procedures across age groups, the limited validation of the instrument in younger children, the absence of current medication data, the lack of repeated measurements, and the absence of a comparison group substantially limit causal and mechanistic interpretation. The study cannot determine how much of the measured anxiety was related to the alleged assault, the judicial and referral process, anticipation of the forensic examination, or pre-existing psychological symptoms. In the absence of a comparison group, the clinical significance of this anxiety burden relative to other paediatric populations likewise cannot be determined.

Nevertheless, the findings indicate that a substantial burden of anxiety symptoms may be present when children arrive for forensic genital examination. This supports the practical value of recognising psychological distress as part of the forensic assessment process, particularly in older adolescents and in children reporting penetration. Such findings should not be interpreted as establishing specific causal pathways, but rather as identifying children who may benefit from timely psychological assessment and access to appropriate support.

Future research should use larger multicentre samples, age-appropriate and validated paediatric anxiety instruments, consistent administration procedures across age groups, repeated assessments before and after forensic evaluation, structured assessment of psychiatric history and current medication use, and appropriate comparison groups. Such designs would allow the temporal and clinical meaning of anxiety symptoms in children undergoing forensic evaluation to be examined more reliably and would provide a stronger basis for determining which characteristics are associated with clinically meaningful psychological distress.

## Figures and Tables

**Table 1:** Anxiety category by age group.

Age Group (Years)	Total n (%)	Minimal n (%)	Mild n (%)	Moderate n (%)	Severe n (%)
6 to 11	14 (13.5)	7 (50.0)	5 (35.7)	2 (14.3)	0 (0.0)
12 to 14	39 (37.5)	8 (20.5)	12 (30.8)	11 (28.2)	8 (20.5)
15 to 17	51 (49.0)	6 (11.8)	9 (17.6)	9 (17.6)	27 (52.9)
Total	104 (100.0)	21 (20.2)	26 (25.0)	22 (21.2)	35 (33.7)

*p* < 0.001 (Fisher–Freeman–Halton exact test). Values are n (row %).

**Table 2:** Anxiety category by sex.

Sex	Total n (%)	Minimal n (%)	Mild n (%)	Moderate n (%)	Severe n (%)
Female	86 (82.7)	14 (16.3)	21 (24.4)	20 (23.3)	31 (36.0)
Male	18 (17.3)	7 (38.9)	5 (27.8)	2 (11.1)	4 (22.2)
Total	104 (100.0)	21 (20.2)	26 (25.0)	22 (21.2)	35 (33.7)

*p* = 0.122 (Fisher–Freeman–Halton exact test). Values are n (row %).

**Table 3:** Anxiety category by educational status.

Educational Status	Total n (%)	Minimal n (%)	Mild n (%)	Moderate n (%)	Severe n (%)
Attending school	68 (65.4)	15 (22.1)	18 (26.5)	19 (27.9)	16 (23.5)
Discontinued education	36 (34.6)	6 (16.7)	8 (22.2)	3 (8.3)	19 (52.8)
Total	104 (100.0)	21 (20.2)	26 (25.0)	22 (21.2)	35 (33.7)

*p* = 0.013 (Pearson’s chi-square test). Values are n (row %).

**Table 4:** Anxiety category by cohabitation status.

Cohabitation Status	Total n (%)	Minimal n (%)	Mild n (%)	Moderate n (%)	Severe n (%)
Both parents	54 (51.9)	15 (27.8)	12 (22.2)	14 (25.9)	13 (24.1)
Mother only	21 (20.2)	3 (14.3)	7 (33.3)	3 (14.3)	8 (38.1)
Father only	6 (5.8)	1 (16.7)	2 (33.3)	1 (16.7)	2 (33.3)
Relatives	10 (9.6)	1 (10.0)	4 (40.0)	1 (10.0)	4 (40.0)
Residential care institution	13 (12.5)	1 (7.7)	1 (7.7)	3 (23.1)	8 (61.5)
Total	104 (100.0)	21 (20.2)	26 (25.0)	22 (21.2)	35 (33.7)

*p* = 0.360 (Fisher–Freeman–Halton exact test). Values are n (row %). Two subgroups are small (six children living with the father only, thirteen in residential care); estimates for these groups are unstable.

**Table 5:** Anxiety category by presence of penetration.

Penetration	Total n (%)	Minimal n (%)	Mild n (%)	Moderate n (%)	Severe n (%)
Present	77 (74.0)	10 (13.0)	13 (16.9)	20 (26.0)	34 (44.2)
Absent	27 (26.0)	11 (40.7)	13 (48.1)	2 (7.4)	1 (3.7)
Total	104 (100.0)	21 (20.2)	26 (25.0)	22 (21.2)	35 (33.7)

*p* < 0.001 (Pearson’s chi-square test). Values are n (row %).

**Table 6:** Anxiety category by penetration type, restricted to the 77 children in whom penetration occurred.

Penetration Type	Total n (%)	Minimal n (%)	Mild n (%)	Moderate n (%)	Severe n (%)	*p*
Vaginal, present	63 (81.8)	6 (9.5)	11 (17.5)	17 (27.0)	29 (46.0)	0.307
Vaginal, absent	14 (18.2)	4 (28.6)	2 (14.3)	3 (21.4)	5 (35.7)	
Anal, present	31 (40.3)	6 (19.4)	6 (19.4)	7 (22.6)	12 (38.7)	0.496
Anal, absent	46 (59.7)	4 (8.7)	7 (15.2)	13 (28.3)	22 (47.8)	
Oral, present	10 (13.0)	0 (0.0)	3 (30.0)	3 (30.0)	4 (40.0)	0.451
Oral, absent	67 (87.0)	10 (14.9)	10 (14.9)	17 (25.4)	30 (44.8)	

**Table 7:** Anxiety category by alcohol or substance influence at the time of the incident.

Alcohol or Substance Influence	Total n (%)	Minimal n (%)	Mild n (%)	Moderate n (%)	Severe n (%)
Present	30 (28.8)	2 (6.7)	5 (16.7)	7 (23.3)	16 (53.3)
Absent	74 (71.2)	19 (25.7)	21 (28.4)	15 (20.3)	19 (25.7)
Total	104 (100.0)	21 (20.2)	26 (25.0)	22 (21.2)	35 (33.7)

*p* = 0.019 (Pearson’s chi-square test). Values are n (row %).

**Table 8:** Anxiety category by history of psychiatric treatment before the incident.

Prior Psychiatric Treatment	Total n (%)	Minimal n (%)	Mild n (%)	Moderate n (%)	Severe n (%)
Present	33 (31.7)	1 (3.0)	6 (18.2)	9 (27.3)	17 (51.5)
Absent	71 (68.3)	20 (28.2)	20 (28.2)	13 (18.3)	18 (25.4)
Total	104 (100.0)	21 (20.2)	26 (25.0)	22 (21.2)	35 (33.7)

*p* = 0.004 (Pearson’s chi-square test). Values are n (row %).

**Table 9:** Anxiety category by recipient of first disclosure.

Recipient of First Disclosure	Total n (%)	Minimal n (%)	Mild n (%)	Moderate n (%)	Severe n (%)
Nuclear family	41 (39.4)	12 (29.3)	14 (34.1)	5 (12.2)	10 (24.4)
Relatives	9 (8.7)	4 (44.4)	4 (44.4)	0 (0.0)	1 (11.1)
Friends	19 (18.3)	3 (15.8)	3 (15.8)	7 (36.8)	6 (31.6)
Healthcare professional	2 (1.9)	0 (0.0)	0 (0.0)	1 (50.0)	1 (50.0)
Teachers	20 (19.2)	1 (5.0)	4 (20.0)	6 (30.0)	9 (45.0)
Law enforcement officers	13 (12.5)	1 (7.7)	1 (7.7)	3 (23.1)	8 (61.5)
Total	104 (100.0)	21 (20.2)	26 (25.0)	22 (21.2)	35 (33.7)

*p* = 0.019 (Fisher–Freeman–Halton exact test). Values are n (row %). Only two children first disclosed to a healthcare professional; this subgroup should not be interpreted individually.

**Table 10:** Anxiety category by perpetrator relationship, physical injury, anogenital findings, previous examination and time to examination.

Variable	Total n (%)	Minimal n (%)	Mild n (%)	Moderate n (%)	Severe n (%)	*p*
**Perpetrator relationship**						0.206
Nuclear family	8 (7.7)	0 (0.0)	3 (37.5)	2 (25.0)	3 (37.5)	
Relative	17 (16.3)	3 (17.6)	2 (11.8)	5 (29.4)	7 (41.2)	
Acquaintance	39 (37.5)	11 (28.2)	9 (23.1)	11 (28.2)	8 (20.5)	
Stranger	18 (17.3)	5 (27.8)	3 (16.7)	3 (16.7)	7 (38.9)	
Contact via social media	21 (20.2)	2 (9.5)	9 (42.9)	1 (4.8)	9 (42.9)	
Two perpetrators	1 (1.0)	0 (0.0)	0 (0.0)	0 (0.0)	1 (100.0)	
**Accompanying physical injury**						0.781
Present	13 (12.5)	3 (23.1)	2 (15.4)	4 (30.8)	4 (30.8)	
Absent	91 (87.5)	18 (19.8)	24 (26.4)	18 (19.8)	31 (34.1)	
**Anogenital findings** (*n* = 77)						0.325
Present	51 (66.2)	4 (7.8)	9 (17.6)	14 (27.5)	24 (47.1)	
Absent	26 (33.8)	6 (23.1)	4 (15.4)	6 (23.1)	10 (38.5)	
**Previous anogenital examination**						0.104
Yes	21 (20.2)	1 (4.8)	4 (19.0)	7 (33.3)	9 (42.9)	
No	83 (79.8)	20 (24.1)	22 (26.5)	15 (18.1)	26 (31.3)	
**Time to examination**						0.579
0 to 7 days	37 (35.6)	6 (16.2)	6 (16.2)	9 (24.3)	16 (43.2)	
8 days to 1 month	13 (12.5)	3 (23.1)	5 (38.5)	2 (15.4)	3 (23.1)	
More than 1 month	54 (51.9)	12 (22.2)	15 (27.8)	11 (20.4)	16 (29.6)	

Values are n (row %). All comparisons tested with the Fisher–Freeman–Halton exact test. Anogenital findings were assessed only in the 77 children in whom penetration occurred.

**Table 11:** Unadjusted and adjusted ordinal logistic regression for anxiety category.

Predictor	Crude OR (95% CI)	*p*	Adjusted OR (95% CI)	*p*
Age group, per one-category increase	3.43 (2.00 to 5.88)	<0.001	1.95 (1.05 to 3.65)	0.035
Discontinued education vs. attending school	2.24 (1.04 to 4.82)	0.040	0.97 (0.41 to 2.31)	0.949
Penetration present vs. absent	8.75 (3.64 to 21.01)	<0.001	5.16 (2.02 to 13.22)	0.001
Alcohol or substance influence vs. none	3.61 (1.61 to 8.11)	0.002	1.88 (0.72 to 4.92)	0.200
Prior psychiatric treatment vs. none	4.04 (1.85 to 8.85)	<0.001	1.28 (0.51 to 3.22)	0.600
First disclosure to a formal recipient vs. informal	4.15 (1.91 to 9.03)	<0.001	2.48 (1.09 to 5.67)	0.031

Proportional-odds ordinal logistic regression, n = 104. Likelihood-ratio chi-square (6 df) = 45.82, *p* < 0.001; McFadden pseudo R-squared = 0.161. Odds ratios above 1 indicate greater odds of falling into a higher anxiety category. A formal recipient denotes a teacher, law-enforcement officer or healthcare professional, as opposed to family, relatives or friends.

## Data Availability

The data presented in this study are available upon reasonable request from the corresponding author. The data are not publicly available because they concern child victims of sexual assault and are subject to judicial and privacy restrictions.

## Supplementary Materials

The following supporting information can be downloaded at https://www.mdpi.com/article/10.3390/diagnostics16193192/s1, Table S1: Sensitivity and robustness analyses of the multivariable model for anxiety category.

## Abbreviations

The following abbreviations are used in this manuscript:

Abbreviation	Definition
aOR	Adjusted odds ratio
BAI	Beck Anxiety Inventory
CI	Confidence interval
cOR	Crude odds ratio
CPS	Child Protective Services
DSM-5	Diagnostic and Statistical Manual of Mental Disorders, Fifth Edition
ICD-11	International Classification of Diseases, 11th Revision
NSPCC	National Society for the Prevention of Cruelty to Children
PTSD	Post-traumatic stress disorder
RCMAS	Revised Children’s Manifest Anxiety Scale
SCARED	Screen for Child Anxiety Related Emotional Disorders
TurkStat	Turkish Statistical Institute
WHO	World Health Organization
