# Supplementary material for: Anxiety Symptom Burden at the Time of Forensic Evaluation in Child Victims of Sexual Assault: A Prospective Study Using the Beck Anxiety Inventory
Source: Diagnostics (Basel). 2026 Oct 1;16(19):3192. doi: 10.3390/diagnostics16193192 (PMC13664563; doi:10.3390/diagnostics16193192)
Supplement: Supplementary file 1 [file diagnostics-16-03192-s001.zip › diagnostics-4513399-supplementary.pdf]

Table S1. Sensitivity and robustness analyses of the multivariable model for anxiety category.

Predictor	Adjusted OR (95% CI)	p-value
A. Main model: proportional-odds ordinal logistic regression (n = 104)		
Age group, per one-category increase	1.95 (1.05 to 3.65)	0.035
Penetration present vs absent	5.16 (2.02 to 13.22)	0.001
First disclosure to a formal recipient	2.48 (1.09 to 5.67)	0.031
B. Sensitivity analysis, participants aged 12–17 years: ordinal logistic regression (n = 90)		
Age group, per one-category increase	2.08 (0.89 to 4.85)	0.090
Penetration present vs absent	5.60 (1.98 to 15.82)	0.001
First disclosure to a formal recipient	2.50 (1.07 to 5.80)	0.033
Penetration, unadjusted within subgroup	7.50 (2.79 to 20.19)	<0.001
C. Bootstrap 95% CIs for the main ordinal model (2000 resamples)		
Age group, per one-category increase	1.95 (1.05 to 4.18)	N/A
Penetration present vs absent	5.16 (2.14 to 15.77)	N/A
First disclosure to a formal recipient	2.48 (1.11 to 6.15)	N/A
D. Binary logistic regression, moderate-to-severe (BAI ≥ 16) vs minimal-to-mild (n = 104)		
Age group, per one-category increase	1.75 (0.77 to 3.98)	0.185
Penetration present vs absent	14.86 (3.37 to 65.55)	<0.001
First disclosure to a formal recipient	4.35 (1.35 to 14.05)	0.014
E. Firth penalised logistic regression, moderate-to-severe vs minimal-to-mild (n = 104)		
Age group, per one-category increase	1.68 (0.77 to 3.79)	0.203
Penetration present vs absent	11.13 (3.22 to 51.72)	0.001
First disclosure to a formal recipient	3.76 (1.31 to 12.21)	0.020
F. Binary logistic regression, severe (BAI ≥ 26) vs non-severe (n = 104)		
Age group, per one-category increase	3.43 (1.31 to 8.99)	0.012
Penetration present vs absent	10.80 (1.25 to 93.23)	0.031
First disclosure to a formal recipient	1.83 (0.65 to 5.14)	0.254

OR, odds ratio; CI, confidence interval; BAI, Beck Anxiety Inventory; N/A, not applicable.

All models were adjusted for the six predictors of the main model (age group, educational status, presence of penetration, alcohol or substance influence at the time of the incident, prior psychiatric treatment, and first disclosure to a formal recipient). Only the three predictors that were significant in the main model are shown; educational status, substance influence and prior psychiatric treatment were non-significant in the main model and remained non-significant in every specification.

A formal recipient denotes a teacher, law-enforcement officer or healthcare professional, as opposed to family, relatives or friends.

Bootstrap intervals are percentile 95% confidence intervals from 2000 resamples; p-values are not defined for percentile bootstrap intervals.

The Firth penalised logistic model (to reduce small-sample bias) and the bootstrap confidence intervals were computed in R (packages logistf and boot); the remaining analyses were performed in IBM SPSS Statistics version 22.0.
